# Steering strategies for wasp inspired self propelled needles

**DOI:** 10.1038/s41598-025-15031-7

**Published:** 2025-08-23

**Authors:** Jette Bloemberg, David Justin Jager, Paul Breedveld, Aimée Sakes

**Affiliations:** 1 Bio-Inspired Technology (BITE) Group, Department of BioMechanical Engineering, Faculty of Mechanical Engineering, Delft University of Technology, Delft, The Netherlands; 2 Department of Electronic and Mechanical Support Division, Faculty of Electrical Engineering, Mathematics and Computer Science, Delft University of Technology, Delft, The Netherlands

**Keywords:** Bioinspired, Design, Needle, Parasitoid wasp, Percutaneous intervention, Biomimetics, Biomedical engineering, Surgery

## Abstract

Positioning a thin needle into a solid substrate near a target region is difficult because the needle can easily bend and buckle. Nevertheless, in nature, female parasitic wasps can do this by using buckling prevention and steering mechanisms. This study presents a self-propelled needle that incorporates wasp-inspired steering mechanisms, specifically, the use of pretension and asymmetry within the needle segments. The needle with an outer diameter of 0.89 millimeters comprises seven parallel needle segments, with the central needle segment being either straight for a forward trajectory or prebent for steering purposes. By retracting and rotating the prebent central needle segment, the needle is capable of omnidirectional steering. The performance of the needle in tissue-mimicking phantoms was evaluated in terms of its propulsion efficiency and steering performance. The propulsion efficiency, affected by slippage of the needle segments with respect to the tissue-mimicking phantoms, was, on average, 63% ± 4% for forward motion and 55% ± 7% for steering motion. Moreover, the needle successfully steered with a mean deflection-to-insertion ratio of 0.41 ± 0.11 (i.e., radius-of-curvature of 44 mm). The proposed bioinspired needle design is a relevant step toward developing steerable needles for percutaneous interventions.

**Supplementary Information:**

The online version contains supplementary material available at 10.1038/s41598-025-15031-7.

## Introduction

From a mechanical standpoint, positioning a thin needle into a solid substrate is difficult because the needle can easily bend and buckle. However, female parasitic wasps do this regularly when they use their long and thin needle-like organ, called the ovipositor, to deposit eggs in a host hidden in wood (e.g., *Megarhyssa atrata* Fabricus (Hymenoptera: Ichneumonidae)^1^ or fruit (e.g., *Diachasmimorpha longicaudata* Ashmead (Hymenoptera: Braconidae)^2^. The wasp ovipositor consists of three parallel segments, called “valves”, which are longitudinally connected by the olistheter mechanism, a jigsaw puzzle-like structure, which allows for sliding of the valves while preventing their separation^3^. The egg channel runs through the center of the three valves^4^. Using its abdominal musculature, the wasp can move the valves longitudinally relative to each other^3^.

The exact mechanisms the parasitic wasp uses for ovipositor insertion and buckling prevention are still being studied. However, Cerkvenik et al.^5^ identified two main mechanisms of ovipositor insertion: (i) pushing the ovipositor into the substrate as a whole, which was observed only in soft substrates, and (ii) inserting with alternating valve movements, which was observed in both soft and solid substrates. In the second mechanism, the wasp moves the valves alternately to achieve a so-called *self-propelled motion*^5,6^. First, one of the valves advances deeper into the tissue, whereas the other valves are held stationary^6,7^. The friction forces of the stationary valves in contact with the surrounding substrate counteract the friction and cutting forces of the advancing valve in contact with the surrounding substrate because of the difference in surface area between the stationary and advancing valves^7^. The wasp alternates the advancing movements between the valves to propel the ovipositor through the substrate incrementally. This alternating valve mechanism enables ovipositor insertion while avoiding net push forces and axial loads that would otherwise result in the wasp being pushed away from the substrate, given the wasp’s small mass, and bending, buckling, or breaking of the ovipositor^5,6^.

**Fig. 1:**
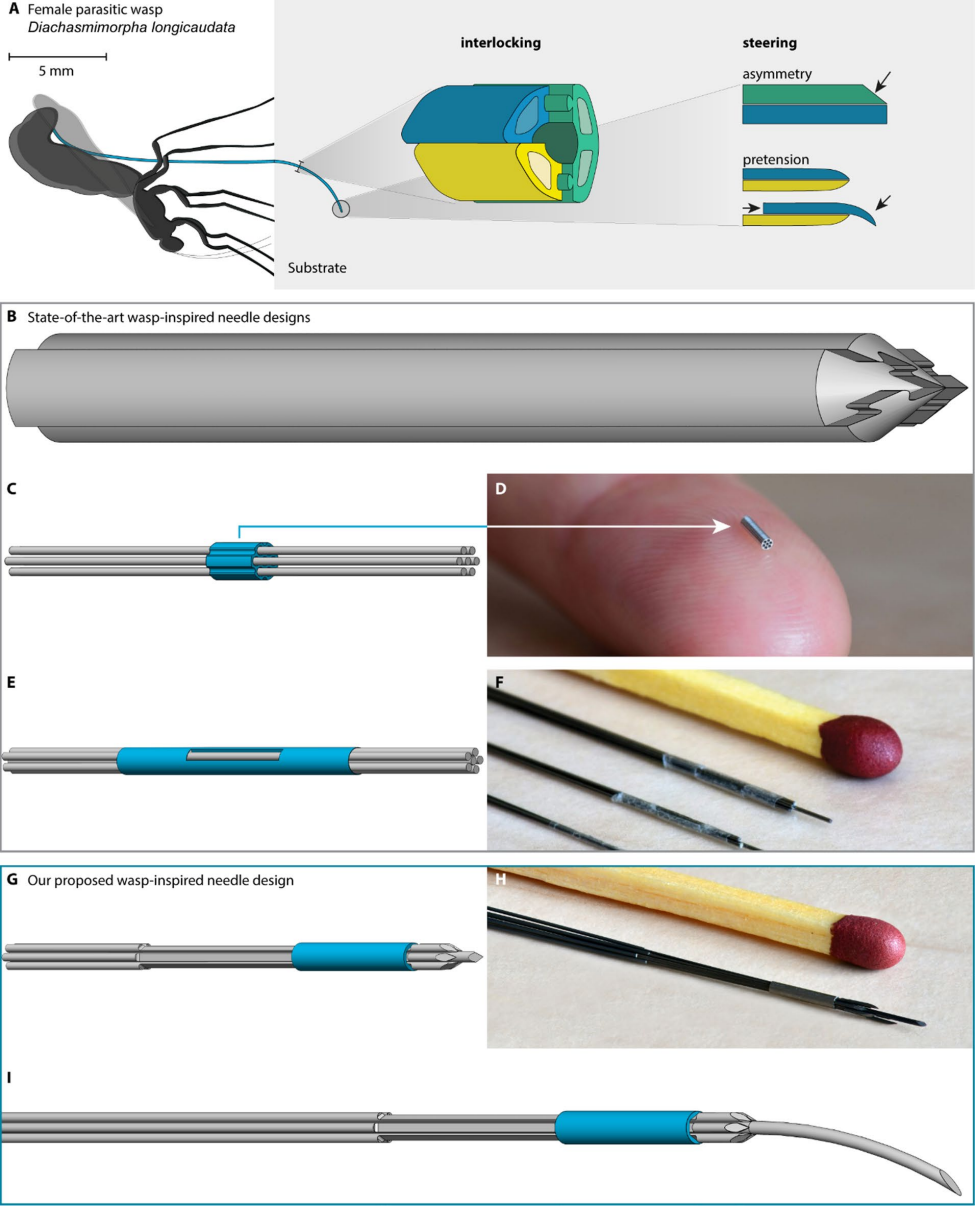
Interlocking and steering in (**A**) female parasitic wasp ovipositors (**B**–**F**), state-of-the-art wasp-inspired needles, and (**G**–**I**) our proposed wasp-inspired needle. (**A**) The ovipositor of female parasitic wasps consists of “valves” (green, yellow, and blue) that are interlocked by the olistheter mechanism, a jigsaw puzzle-like structure. The wasps are hypothesized to steer their ovipositor by asymmetry and pretension of their ovipositor valves (based on Cerkvenik et al.^5^. (**B**) Four wedge-shaped sections with a puzzle-like interlocking mechanism that mimics the wasp’s olistheter mechanism (based on Burrows et al.^27^. (**C**) Flower-shaped interlocking ring (blue) around seven needle segments (gray) (based on Scali et al.^14^, (**D**) photo of the interlocking ring (from^14^. (**E**) Ultrathin-walled heat shrink tube (blue) that bundles six needle segments (gray) (based on Scali et al.^13^, (**F**) photo of several needle segment tips with the heat shrink tube (from^13^. (**G**) Our proposed needle consists of six needle segments with indentations (gray), a hexagonal interlocking ring (blue), and a central needle segment (gray), (**H**) photo of our proposed needle, (**I**) our proposed needle in the steering configuration with a prebent bevel-shaped central needle segment.

In addition to ovipositor insertion along a straight path without buckling, the female parasitic wasp can also curve and steer its ovipositor to reach the desired target (Fig. 1A)^5^. In the scientific literature, several hypotheses attempt to explain the steering mechanism of the wasp ovipositor. According to one prevalent hypothesis, a wasp can steer its ovipositor because of its asymmetric beveled tip^5^. When moving such an asymmetric beveled tip through a substrate, the off-axis reaction forces applied by the substrate on the tip cause the ovipositor to bend^8^, resulting in a curved path^9^. Moreover, the bevel shape can presumably be enhanced by changing the relative position of the valves, creating an offset at the tip in the required direction. On the other hand, it is hypothesized that the valves of the ovipositor exhibit pretension and tend to curve to one side when not opposed by the other valves^5^. When the valves are aligned with their tips, the pretensions in the valves counteract each other, resulting in a straight structure. In contrast, when a valve protrudes, its tip curves inward toward the other valves. For both ovipositor steering hypotheses, the ability to steer offers a means to control the ovipositor trajectory during insertion.

Inspired by the ovipositor of the parasitic wasps, a variety of self-propelled and steerable needles consisting of multiple parallel needle segments have been proposed in the scientific literature^10–20^. The self-propelled motion in these needles is achieved by advancing a smaller number of needle segments than the number of stationary needle segments, thereby counterbalancing the cutting and friction force of the advancing needle segments by the friction force generated by the remaining stationary segments^19^. Research has shown that tissue motion and damage in the needle vicinity are reduced when a multisegmented needle actuated with a reciprocal advancing motion is used as compared to merely pushing the needle through the tissue^10,11,21^.

Inspired by the bevel shape of the wasp ovipositor, omnidirectional steering of wasp-inspired needles was reported in the scientific literature by inducing an offset between the needle segments, creating a discrete bevel-shaped tip^10,12,14,20^. However, the steering curvature achieved with bevel-shaped needles is limited and dependent on the needle-tissue interaction forces^22^. We hypothesize that implementing prebending in the needle, similar to the pretension in the ovipositor of the parasitic wasp, could sharpen the steering curvature. A comparable steering strategy has been successfully applied in concentric tube needles using precurved outer tubes and inner wires^23^. However, these tubes are not capable of a self-propelled motion to prevent needle buckling.

For the wasp-inspired self-propelling and steering mechanisms to work, the needle segments must be interlocked at the tip to avoid separation. A common interlocking mechanism described in the scientific literature mimics the ovipositor’s olistheter mechanism (Fig. 1B)^10,20,24–28^. For example, Burrows et al.^27^ and Aktas et al.^28^ developed 3D-printed 4-mm and thermally-drawn 1.3-mm diameter needles, respectively, consisting of wedge-shaped parallel needle segments, which slide alongside one another, using jigsaw puzzle-like structures that interlock the needle segments along their entire length. A disadvantage of this interlocking mechanism is that it is difficult to miniaturize. In order to miniaturize the needle diameter to submillimeter dimensions, Scali et al.^12,14^. stepped away from the wasp-inspired interlocking mechanism and used a flower-shaped ring as the interlocking mechanism (Fig. 1C,D). However, the increased cross-sectional area caused by the flower-shaped ring allows for potential tissue accumulation between the needle segments and increases the cutting forces, thereby hindering the self-propelled motion of the needle. Furthermore, the flower-shaped ring does not constrain the axial rotation of individual needle segments. Needle segment rotation is not a problem when we use blunt needle segments. However, when we use bevel-shaped needle segments to sharpen the needle or to allow for steering, rotation of the needle segments can cause these segments to misalign and not point toward the center. This misalignment may lead to divergence of the bevel-shaped segments and potentially allow tissue accumulation between them.

In follow-up designs, Scali et al.^13^ and Bloemberg et al.^15,16,29^ replaced the flower-shaped ring with an ultrathin-walled heat shrink tube as the interlocking mechanism (Fig. 1E,F). The heat shrink tube is glued to one of the needle segments to bundle the needle segments at the tip. This solution only minimally increases the needle diameter. However, the heat shrink tube does not constrain the axial rotation of the needle segments.

Previous research has demonstrated the efficacy of using wasp-inspired mechanisms in the development of self-propelled and steerable needles^10,12–16,20,24–29^. To date, their steering capabilities have been attained by mimicking the asymmetric bevel shape of the wasp ovipositor. Furthermore, the parallel needle segments were interlocked via a hard-to-miniaturize jigsaw puzzle-like structure, a flower-shaped ring that hinders the self-propelled motion of the needle, or a heat shrink tube that does not constrain axial rotation.

This study explores an alternative steering system inspired by the parasitic wasp complemented by a novel interlocking mechanism. The steering system incorporates prebending and asymmetry within the centrally positioned needle segment (Fig. 1G–I). Our novel interlocking mechanism constrains axial rotation of the needle segments without locally increasing the overall needle diameter. Accordingly, we present a submillimeter needle suitable for insertion via a self-propelled motion and omnidirectional steering.

## Results

### Needle design

#### Interlocking

In this study, we present a new design of a submillimeter-diameter self-propelled steerable needle, the Prebent Ovipositor Needle, with an improved interlocking mechanism. The Prebent Ovipositor Needle has an outer diameter of 0.89 mm, making it suitable for percutaneous interventions. To prevent separation of the needle segments at the tip, our interlocking mechanism consists of a hexagonal interlocking ring (in blue) and six outer needle segments (in gray) arranged in a circle around a central needle segment (in gray) (Fig. 1G,H). The outer needle segments contain lancet-shaped tips that face inward toward the central needle segment, whereas the central needle segment has a triangular sharp tip, resulting in an assembled needle with a symmetric sharp tip (Fig. 1G). Additionally, the outer needle segments contain indentations that leave a semicircular cross-section over a distance of 12 mm. The interlocking ring fits around the needle segments at the position of the indentations. As a result, the wall thickness of the interlocking ring is equal to the radius of the outer needle segments, ensuring that the interlocking ring neither enlarges the overall needle diameter nor allows for potential tissue accumulation between the needle segments.

Our interlocking ring design ensures that the lancet-shaped tips of the outer needle segments always point toward the central needle segment. The length of the interlocking ring is shorter than the length of the indentations, allowing relative translation of the six outer needle segments along the axial direction to initiate wasp-inspired self-propelled motion. The indentations allow needle assembly through two sequential steps: (i) individually threading the outer needle segments through the interlocking ring and (ii) guiding the central needle segment through the center to align the indentations of the outer needle segments with the internal surfaces of the interlocking ring.

#### Self-propelling

The resulting Prebent Ovipositor Needle design enables a self-propelled motion through the relative movement of the outer needle segments. This self-propelled motion is achieved by counterbalancing the cutting and friction forces perceived by the advancing segments with the friction force generated by the stationary segments^19^. For effective self-propulsion, the friction forces between the substrate and the stationary needle segments must be equal to the combined friction and cutting forces perceived by the advancing needle segments. Consequently, we decided on a motion sequence where one outer needle segment advances while five outer needle segments remain stationary with respect to the substrate. This motion sequence can be actuated by a control and actuation system.

**Fig. 2:**
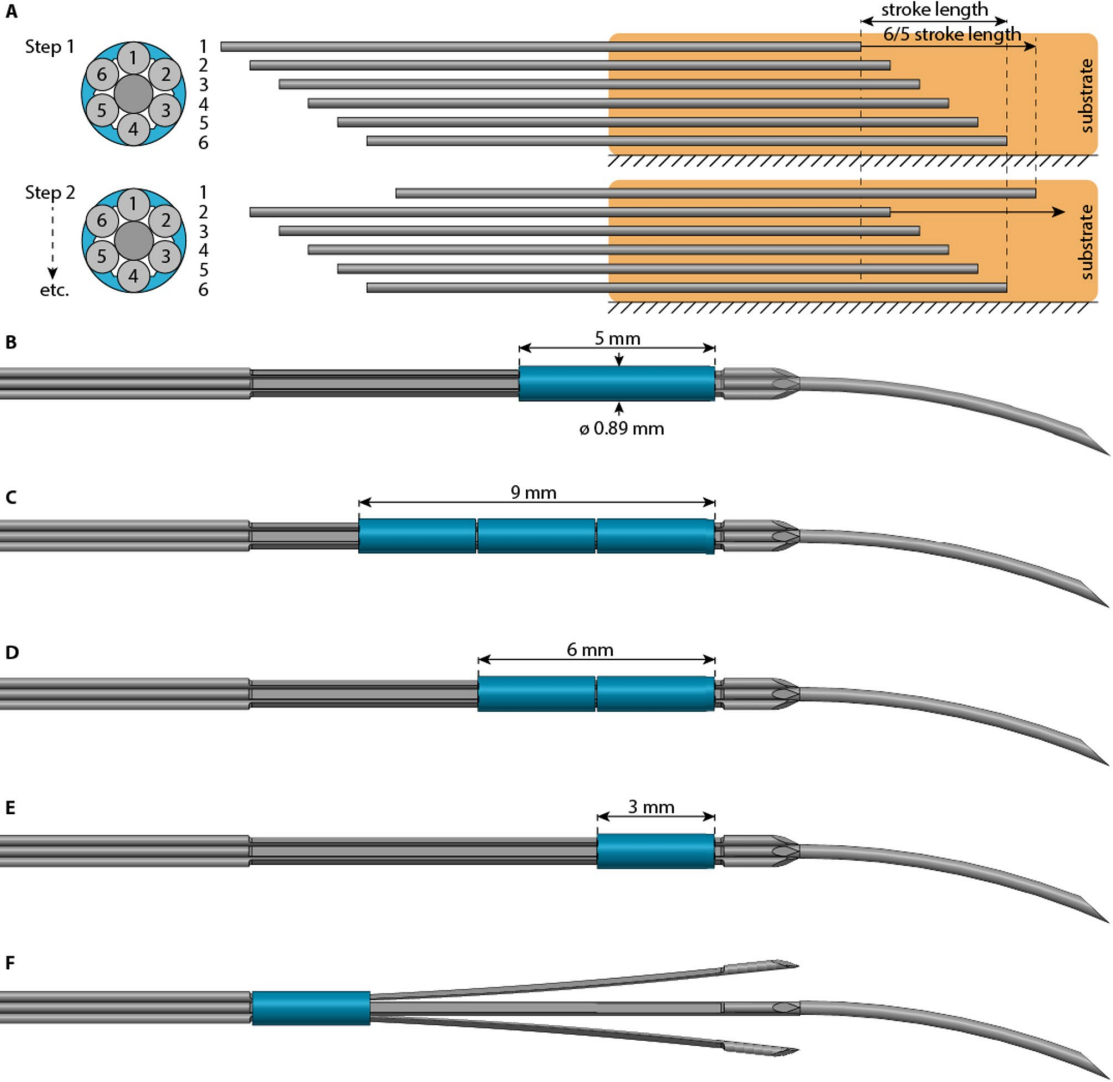
Self-propelled steerable needle tip designs. (**A**) Self-propelled motion sequence: during Steps 1–6 of the actuation cycle, one outer needle segment moves forward over $\:\frac{6}{5}$ stroke length with respect to the surrounding substrate (orange). (**B**) A single interlocking ring measuring 5 mm in length. (**C–E**) Three, two, and one interlocking ring(s) measuring 3 mm in length each. (**F**) Divergence of the outer needle segments with a single 3-mm interlocking ring.

The needle’s self-propelled motion relies on sequentially translating the six outer needle segments in seven steps per *actuation cycle*. During *Steps 1–6* (Fig. 2A), one outer needle segment is advanced by a defined distance of $\:\frac{6}{5}$ *stroke length* with respect to the substrate, whereas the other five outer needle segments remain stationary relative to the substrate. *Step 7* of the actuation cycle is a *reset step*, in which the interlocking ring is moved forward to the tip of the needle segments by consecutively advancing and retracting one of the outer needle segments. When there is no slippage of the stationary needle segments with respect to the substrate, the needle self-propels step by step into the substrate.

#### Steering

The parasitic wasp is hypothesized to steer its ovipositor through the pretension and asymmetry in its valves^5^. We decided to implement these mechanisms in our Prebent Ovipositor Needle. The central needle segment can be either a straight triangular needle segment (Fig. 1G) or a prebent bevel-shaped needle segment (Fig. 1I). When a central needle segment with a triangular sharp tip is used, the needle follows a straight trajectory during insertion into a substrate. In contrast, using the prebent bevel-shaped needle segment enables the needle to steer. The curvature of the prebent influences the extent of needle bending and the sharpness of the generated curve. By axially rotating the central needle segment, the steering direction can be changed to achieve omnidirectional steering.

Multiple versions of the interlocking ring were developed throughout the design process to optimize the interlocking mechanism for both the self-propelled motion and the steering motion. The initial design featured an interlocking ring with a length of 5 mm (Fig. 2B, referred to as *1R5* from hereon). In the other designs, we split the 12-mm indentation length of the outer needle segments into four parts of equal length. Consequently, we opted to develop a range of configurations, including three interlocking rings of 3 mm each (Fig. 2C, referred to as *3R3* from hereon), resulting in a minimal stroke length of 3 mm, two 3-mm interlocking rings (Fig. 2D, referred to as *2R3* from hereon), and a single 3-mm interlocking ring (Fig. 2E,F, referred to as *1R3* from hereon). This variety allowed us to evaluate the balance between stability and flexibility within the needle design during both the self-propelled and steering motion.

### Performance evaluation

#### Self-propelling and steering

For the performance evaluation, we assessed the performance of the Prebent Ovipositor Needle in soft tissue-mimicking phantoms made of gelatin with a gelatin weight ratio (wt) of 5 wt%. The use of 5-wt% gelatin results in a modulus of elasticity of 5.3 kPa as determined by Scali et al.^13^, this modulus of elasticity approximates healthy liver tissue (< 6 kPa)^30^. The performance of the Prebent Ovipositor Needle was evaluated based on its propulsion and steering performance. The propulsion performance was quantified in terms of the propulsion efficiency ($\:\eta\:$ [%]), slip ratio ($\:{s}_{\text{r}}$ [-]), and insertion speed ($\:v$ [mm/s]):

1 $$\:\varvec{\eta\:}=\frac{{\varvec{d}}_{\mathbf{m}}}{{\varvec{d}}_{\mathbf{e}}}\:\bullet\:100\varvec{\%}$$

2 $$\:{\varvec{s}}_{\mathbf{r}}=1-\frac{{\varvec{d}}_{\mathbf{m}}}{{\varvec{d}}_{\mathbf{e}}}$$

3 $$\:\varvec{v}=\frac{{\varvec{d}}_{\mathbf{m}}}{\varvec{t}}$$

where $\:{d}_{\text{m}}$ [mm] is the measured insertion distance of the needle with respect to the tissue-mimicking phantom, $\:{d}_{\text{e}}$ [mm] is the expected insertion distance, and $\:t$ [s] is the measured insertion time. For $\:\eta\:=100\%$ and $\:{s}_{\text{r}}=1$, the advancing needle segment moves forward into the tissue-mimicking phantom while the remaining needle segments remain stationary relative to the tissue-mimicking phantom, meaning that there is no slip between stationary needle segments and the tissue-mimicking phantom, creating a forward needle motion at maximum speed. Variable $\:{d}_{\text{e}}$ was calculated via Eq. (4):

4 $$\:{d}_{\text{e}}=\frac{6}{5}\bullet\:S\bullet\:C$$

where $\:S$ [mm] is the stroke length, which was set to 2 mm, and $\:C$ [–] is the number of actuation cycles, which was set to 25, resulting in $\:{d}_{\text{e}}=60$ mm; for more details on the expected insertion distance, see the Supplementary Materials (Figs. S1-S3). The 2-mm stroke length, approximately twice the needle’s 0.89-mm diameter, facilitates incremental movement of the outer needle segments through the substrate while following the central needle segment trajectory and minimizing the risk of needle segment buckling. The number of actuation cycles is constrained by the total needle length and the experimental setup.

To quantify the steerability of the needle, we evaluated the measured straight insertion distance, $\:{d}_{\text{s}}$, and the deflection of the needle from a straight path, $\:{d}_{\text{d}}$. The ratio of distance $\:{d}_{\text{d}}$ to distance $\:{d}_{\text{s}}$ is called the deflection-to-insertion ratio ($\:{d}_{\text{r}}$ [–]) and was calculated via Eq. (5):

5 $$\:{d}_{\text{r}}=\frac{{d}_{\text{d}}}{{d}_{\text{s}}}$$

where $\:{d}_{\text{d}}$ [mm] represents the difference between the needle’s initial and final distances relative to its centerline. These distances were measured as the deviation of the needle tip perpendicular to the imaginary straight line that the needle would have followed if it had not been steered. Besides $\:{d}_{\text{r}}$, the needle’s steerability was quantified by its achieved curvature ($\:\kappa\:$ [mm^− 1^]), which is the amount by which a curve deviates from a straight line. In the case of a circle, the curvature is the reciprocal of its radius. To calculate $\:\kappa\:$, we approximated the needle’s final trajectory as a segment of a circle with a radius $\:R$ [mm] that was fitted to the needle’s final trajectory via Eq. (6):

6 $$\:\kappa\:=\frac{1}{R}=\frac{2{d}_{\text{d}}}{{d}_{\text{s}}^{2}+{d}_{\text{d}}^{2}}$$

Variables $\:\kappa\:$ and $\:{d}_{\text{r}}$ are large when the radius of curvature is small, indicating that the needle makes a sharp curve. Both $\:{d}_{\text{r}}=0$ and $\:\kappa\:=0$ mm^−1^ indicate a straight needle insertion trajectory.

**Fig. 3:**
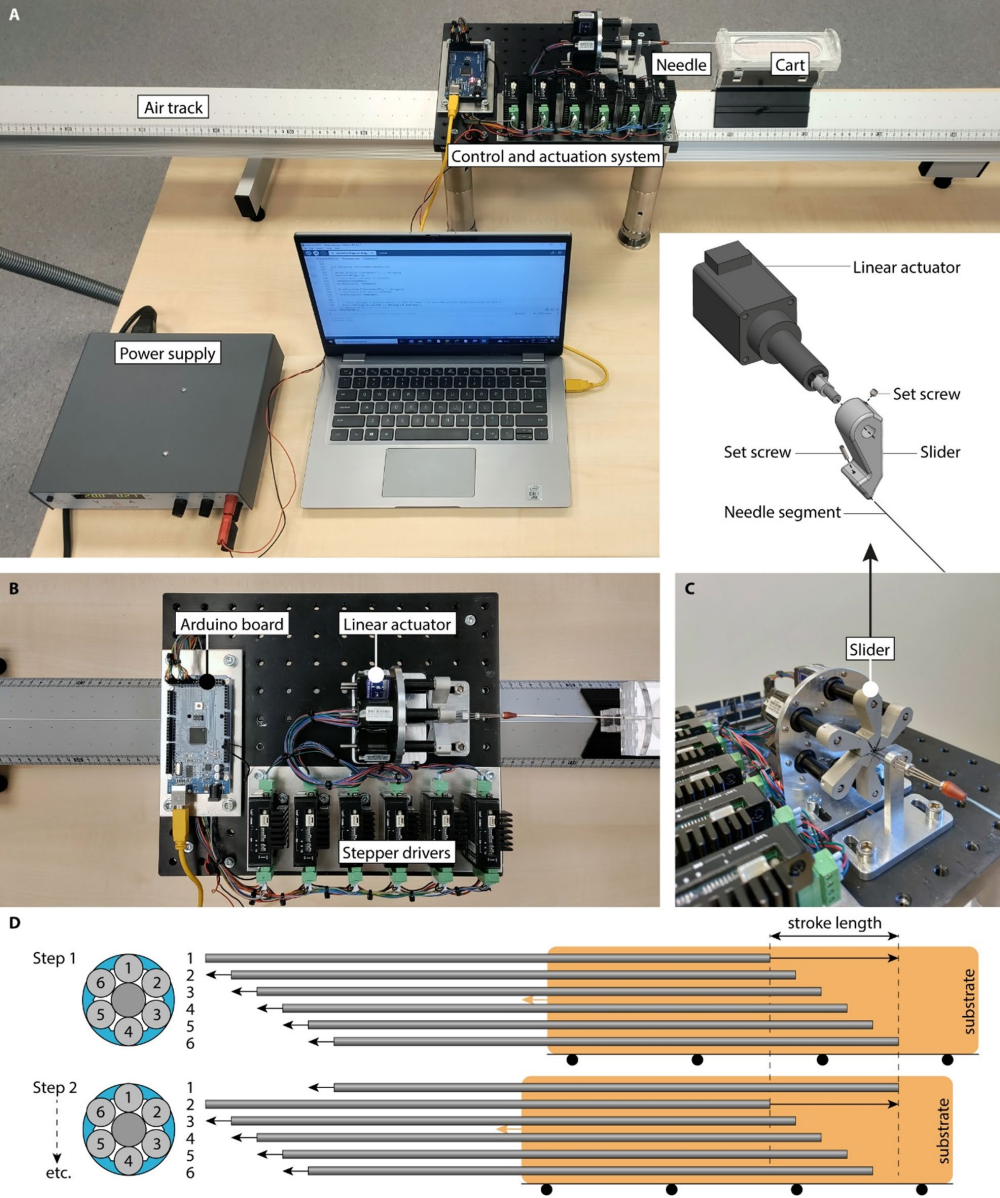
Experimental setup for the evaluation of the Prebent Ovipositor Needle performance. (**A**) The control and actuation system was mounted on an aluminum base plate. The needle, connected to the control and actuation system, is propelled and steered into a tissue-mimicking phantom carried by a cart on an air track. (**B**) Top view of the control and actuation system comprising an Arduino board, six stepper drivers, and six linear actuators. (**C**) Each linear actuator and outer needle segment was clamped to a slider using set screws. (**D**) Self-propelled motion sequence: during Steps 1–6 of the actuation cycle, one outer needle segment moves forward over the stroke length while the other outer needle segments move slowly backward over one-fifth of the stroke length thereby self-propelling the needle step by step into the substrate by pulling the substrate (orange) on the cart on the air track (illustrated by black wheels) toward the control and actuation system.

The experimental setup consisted of the Prebent Ovipositor Needle connected to a control and actuation system, mounted to a solid aluminum breadboard (MB2530/M, Thorlabs, Inc., Newton, NJ), and a tissue-mimicking phantom placed on a customized Perspex cart on an air track (Eurofysica, ‘s-Hertogenbosch, The Netherlands) (Fig. 3A). Rather than moving the needle toward the tissue-mimicking phantom, we chose to move the tissue-mimicking phantom toward the stationary needle. This setup involved keeping the needle stationary and connected to the control and actuation system that facilitates the relative motion of the needle segments for the self-propelled motion (Figs. 3B,C). The tissue-mimicking phantom was placed on the cart on the air track to allow a near-frictionless horizontal translation of the tissue-mimicking phantom. The Prebent Ovipositor Needle self-propels if it pulls the tissue-mimicking phantom toward the control and actuation system by pulling itself deeper into the tissue-mimicking phantom (Fig. 3D).

**Fig. 4:**
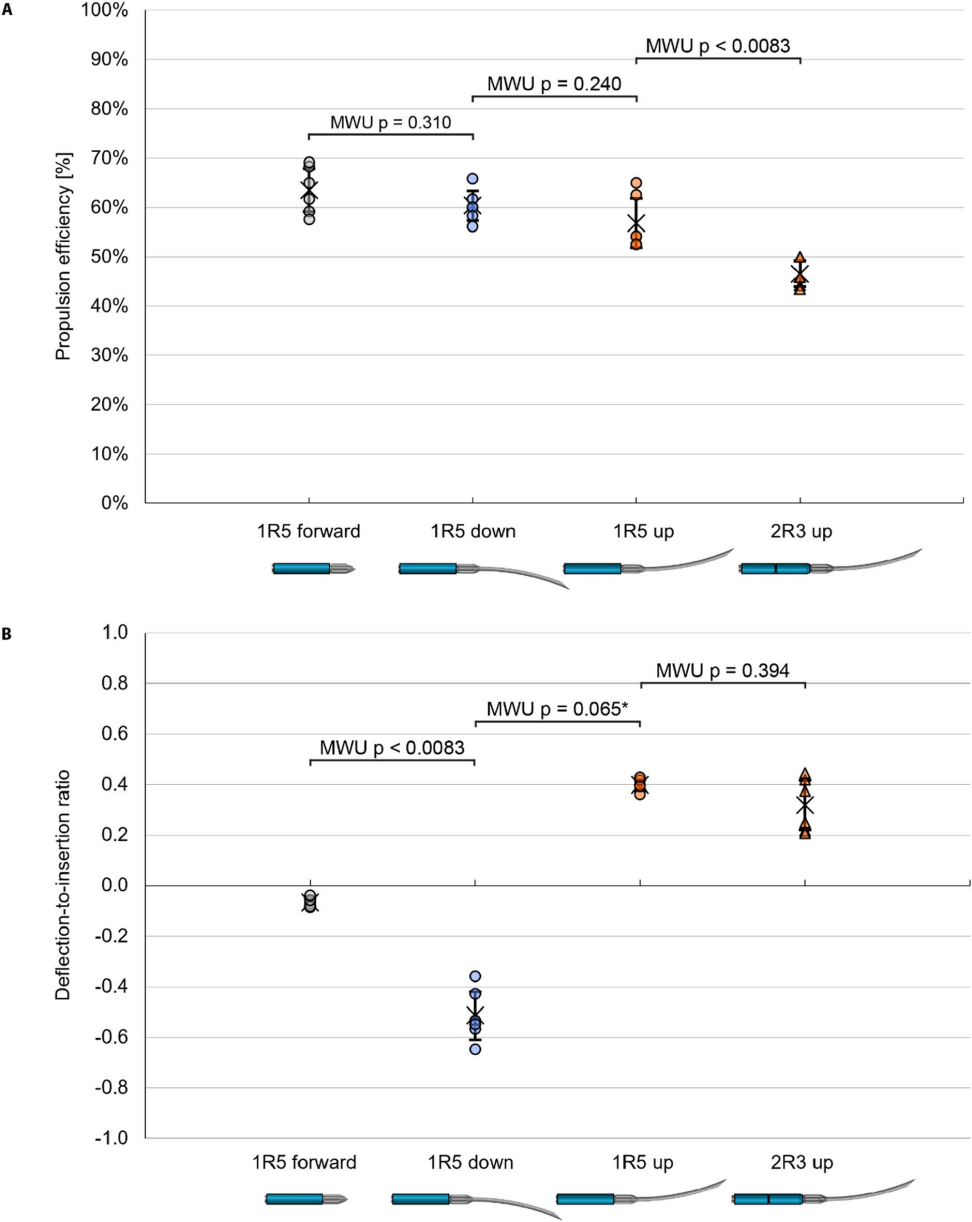
Experimental results of (**A**) propulsion and (**B**) steering performance evaluations of the Prebent Ovipositor Needle in a 5-wt% gelatin phantom mimicking healthy liver tissue. The circles represent single trials, the crosses represent mean values, and the error bars represent the standard deviation. A positive deflection-to-insertion ratio corresponds to steering upward, and a negative deflection-to-insertion ratio corresponds to steering downward. Meaning of abbreviations of the different conditions shown at the x-axes: 1R5 forward = straight triangular central segment and a single 5-mm interlocking ring, 1R5 down = prebent bevel-shaped central segment steering downward and a single 5-mm interlocking ring, 1R5 up = prebent bevel-shaped central segment steering upward and a single 5-mm interlocking ring, and 2R3 up = prebent bevel-shaped central segment steering upward and two 3-mm interlocking rings. Annotations in the figure denote the p-values of the Mann–Whitney U (MWU) test. A p-value less than 0.0083 indicates a significant difference. *The Mann-Whitney U test was performed on the absolute values of $\:{d}_{\text{r}}$ between the 1R5 up and 1R5 down conditions.

**Fig. 5:**
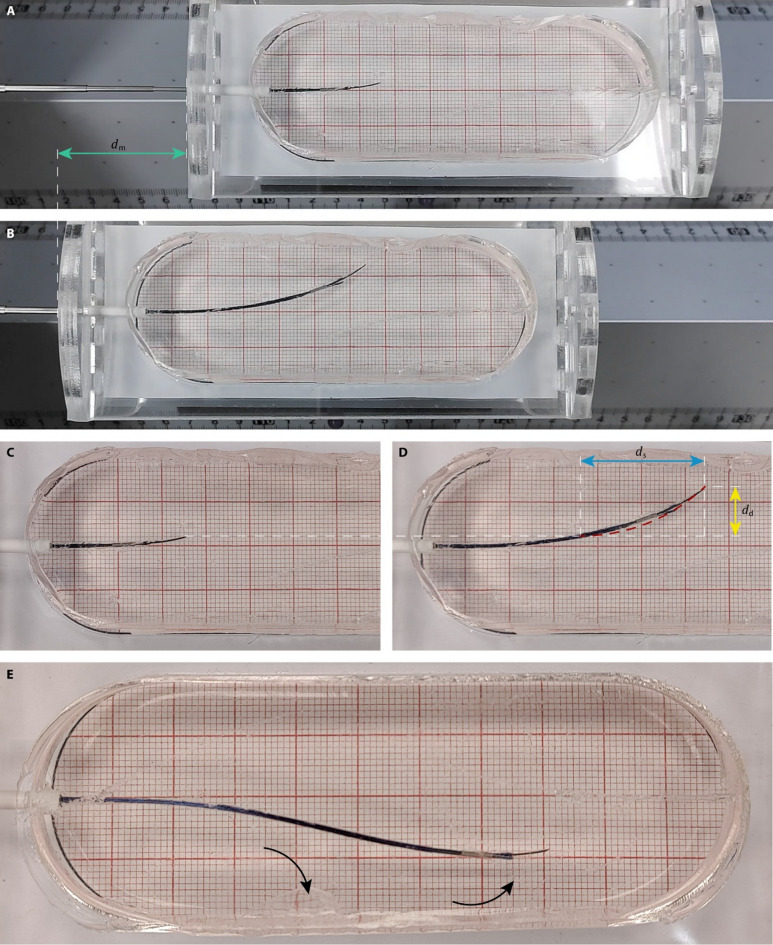
Images of the needle inside the gelatin-based tissue-mimicking phantom. Images of a needle with a single 5-mm interlocking ring steering upward: (**A**) the initial frame and (**B**) the final frame showing variable $\:{d}_{\text{m}}$ [mm] (green), the measured insertion distance of the needle with respect to the tissue-mimicking phantom. Close-ups of (**C**) the initial frame and (**D**) the final frame showing variables $\:{d}_{\text{s}}$ [mm] (blue), the measured straight insertion distance, and $\:{d}_{\text{d}}$ [mm] (yellow), the deflection of the needle from a straight path, and an overlay of the computed curvature (red dashed). (**E**) Final position of the needle after changing the steering direction. The arrows show the direction of steering. The steering direction changed from downward to upward.

We conducted three consecutive experiments to evaluate the effects of (1) the central needle design, (2) the steering direction, and (3) the interlocking ring configuration on the performance of the Prebent Ovipositor Needle (Supplementary Video 1). The results for $\:\eta\:$, $\:{s}_{\text{r}}$, $\:v$, $\:{d}_{\text{r}}$, and $\:\kappa\:$ are summarized in Table 1; Fig. 4 and example measurements of variables $\:{d}_{\text{m}}$, $\:{d}_{\text{s}}$, $\:{d}_{\text{d}}$, and $\:\kappa\:$ are visualized in Fig. 5A–D. First, we compared the downward-steering prebent bevel-shaped central needle design (1R5 down) to the straight triangular central needle design (1R5 forward). No statistically significant difference was found for $\:\eta\:$ between the 1R5 forward (median = 63%) and 1R5 down (median = 60%) conditions (U = 11.5, *p* = 0.310), indicating no statistically significant difference in propulsion efficiency between straight and steering trajectories. However, the 1R5 down condition (median = − 0.54) showed a significantly difference in $\:{d}_{\text{r}}$ as compared to the 1R5 forward condition (median = −0.07, U = 0, *p* = 0.002), indicating effective downward steering.

Second, we evaluated whether the steering performance was consistent across different steering directions by comparing the propulsion and steering performance when the prebent bevel-shaped central segment was directed upward (1R5 up) and downward (1R5 down). No statistically significant difference was found for $\:\eta\:$ between the 1R5 up (median = 54%) and 1R5 down (median = 60%) conditions (U = 10, *p* = 0.240). Furthermore, no statistically significant difference was found for the absolute $\:{d}_{\text{r}}$ between the 1R5 up (median = 0.40) and 1R5 down (median = 0.54) conditions (U = 6.5, *p* = 0.065). These results indicate no statistically significant differences in either propulsion or steering performance for equal prebents in different steering directions.

Finally, we investigated the impact of different interlocking ring configurations by comparing the 1R5, 3R3, 2R3, and 1R3 designs. Since the second experiment demonstrated that the steering direction did not affect the propulsion and steering performance, we focused solely on the effects of the interlocking ring design in a single steering direction: upward steering. The 3R3 configuration (Fig. 2C) hindered needle advancement within the tissue-mimicking phantom due to its 9-mm interlocking ring length. During the reset step (Step 7), the overall friction force perceived by the three interlocking rings moving forward as a single, 9-mm long unit exceeded the friction between the five stationary outer needle segments and the substrate, causing the stationary needle segments to be pushed backward and start slipping relative to the substrate. The 1R3 configuration (Fig. 2E) led to divergence of the needle segments within the tissue-mimicking phantom (Fig. 2F) because the 3-mm ring allows the needle segments to protrude by 11 mm beyond the ring. This divergence strongly increases the force required to advance the ring over the needle segments, ultimately blocking the needle’s advancement within the substrate. Therefore, the 3R3 and 1R3 designs are not feasible for a self-propelled steerable motion. A statistical analysis of the remaining interlocking ring configurations, 1R5 (Fig. 2B) and 2R3 (Fig. 2D), resulted in the 1R5 up condition (median = 0.40) showing a statistically significant difference in $\:\eta\:$ as compared to the 2R3 up condition (median = 0.31, U = 0, *p* = 0.002). However, no statistically significant difference was found for $\:{d}_{\text{r}}$ between the 1R5 up (median = 0.40) and 2R3 up (median = 0.31) conditions (U = 12, *p* = 0.394). These results suggest an advantage of the single 5-mm ring design in terms of the propulsion performance over the double 3-mm ring design, while no statistically significant difference in steering performance was found.

**Table 1:**
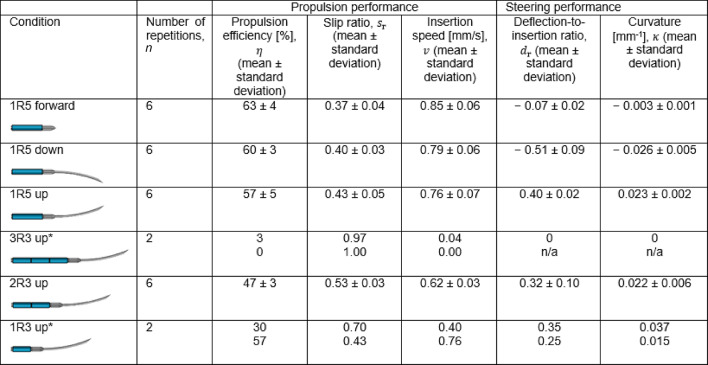
Experimental results of self-propelling and steering performance evaluations of the Prebent Ovipositor Needle in a 5-wt% gelatin phantom mimicking healthy liver tissue, showing the condition, number of repetitions (i.e., how often the condition was evaluated in a new gelatin phantom), propulsion efficiency, slip ratio, insertion speed, deflection-to-insertion ratio, and curvature, with mean values and standard deviations. A positive deflection-to-insertion ratio and curvature correspond to upward steering, and a negative deflection-to-insertion ratio and curvature correspond to downward steering. Meaning of abbreviations of the different conditions: 1R5 forward = straight triangular central segment and a single 5-mm interlocking ring, 1R5 down = prebent bevel-shaped central segment steering downward and a single 5-mm interlocking ring, 1R5 up = prebent bevel-shaped central segment steering upward and a single 5-mm interlocking ring, 3R3 up = prebent-bevel-shaped central needle segment steering upward and three 3-mm interlocking rings, 2R3 up = prebent bevel-shaped central segment steering upward and two 3-mm interlocking rings, and 1R3 up = prebent bevel-shaped central needle segment steering upward and a single 3-mm interlocking ring.

*For the 3R3 and 1R3 conditions, the values of individual repetitions are provided instead of mean ± standard deviation, as the conditions were only evaluated twice.

#### Explorative evaluation

We conducted a set of additional tests to explore the behavior of the Prebent Ovipositor Needle in different settings. First, we performed a qualitative test to evaluate the ability of the needle to change its steering direction during insertion in a 5-wt% gelatin phantom mimicking healthy liver tissue. In this test, the needle segments were actuated over twenty actuation cycles while steering downward, followed by another twenty actuation cycles steering upward. The steering direction was changed by slightly retracting the central needle segment, axially rotating it over 180°, and slightly advancing it again. The measurement revealed that the needle was able to change direction during the self-propelled insertion (Fig. 5E, Supplementary Video 2).

Second, we investigated the behavior of the Prebent Ovipositor Needle in its 1R5 down and 1R5 up configurations in terms of its propulsion and steering performance using a shorter stroke length, $\:S$, of 1 mm instead of 2 mm. For each configuration, we conducted two tests over 50 actuation cycles. The results revealed propulsion efficiency, $\:\eta\:$, percentages of 38% and 44–45% for the 1R5 down and 1R5 up conditions, respectively. Although these efficiencies are comparable to each other, they are smaller than those achieved for $\:S=2$ mm, with mean $\:\eta\:$ percentages of 60% ± 3% and 57% ± 5%, respectively. These preliminary results suggest that the needle may propel more efficiently when actuated at a longer stroke length, $\:S$, as compared to a shorter stroke length. However, further research with additional stroke lengths is required to confirm this trend. The deflection-to-insertion ratio, $\:{d}_{\text{r}}$, values for $\:S=1$ mm were −0.58 to −0.54 for the 1R5 down condition and 0.41 to 0.7 for the 1R5 up condition, both of which are comparable to those achieved for $\:S=2$ mm, with mean $\:{d}_{\text{r}}$ values of −0.51 ± 0.09 and 0.40 ± 0.02, respectively. This suggests that the needle steers with equal efficiency regardless of whether it is actuated at longer or shorter stroke lengths.

Finally, we investigated the behavior of the Prebent Ovipositor Needle in its 1R5 forward configuration in stiffer substrates. For this purpose, we used 10-wt% gelatin for the tissue-mimicking phantoms, which resulted in a modulus of elasticity of 17 kPa as determined by Scali et al.^13^, approximating healthy prostate or cirrhotic liver tissue^30,31^. We conducted two tests with the 1R5 forward configuration actuated with stroke length, $\:S$, of 2 mm and number of actuation cycles, $\:C$, of 25. The results revealed that the Prebent Ovipositor Needle self-propelled through the stiff substrate with a propulsion efficiency, $\:\eta\:$, of 52–58% and deflection-to-insertion ratio, $\:{d}_{\text{r}}$, of −0.05 to 0.00. Both the measured propulsion efficiency, $\:\eta\:$, and deflection-to-insertion ratio, $\:{d}_{\text{r}}$, in the 10-wt% tissue-mimicking phantoms are slightly smaller than those reported for the 5-wt% tissue-mimicking phantoms, which are 63% ± 4% and −0.07 ± 0.02 (Table 1), respectively. This indicates that the needle propels slightly faster and steers with slightly sharper curves in soft substrates as compared to stiff substrates. The reduced propulsion efficiency in stiff substrates can be attributed to the increased cutting force, which arises from the plastic deformation of the substrate and the resistance due to the substrate stiffness at the needle tip^32^.

## Discussion

In contrast to previously developed wasp-inspired self-propelled and steerable needles that rely on inducing an offset between the needle segments to create a discrete bevel-shaped tip for steering^10,12,14,20^, we presented a steerable needle that incorporates an alternative steering mechanism inspired by the parasitic wasp, specifically, the use of prebending combined with a bevel-shaped tip within the needle segments. Additionally, our needle features a new needle interlocking mechanism that constrains needle segment rotation. Our needle comprises seven parallel needle segments, with the central needle segment tip being either straight and triangular for a forward trajectory, or prebent and bevel-shaped for steering purposes. By rotating the prebent needle segment, our needle is capable of omnidirectional steering, allowing for trajectory changes without the need for axial rotation of the entire needle. Changing the steering direction without axial rotation prevents the generation of torsional stress by the surrounding tissue on the needle, thereby preventing angular lag between the orientation of the needle base and tip^33^. Consequently, our prebending steering method can enhance the controllability of the needle trajectory while potentially reducing the risk of tissue damage as compared to steerable needles that require axial rotation of the entire needle^34^.

In our data analysis, we performed statistical comparisons with non-parametric tests and corrections for multiple comparisons. However, the low number of repetitions per condition (i.e., 6) is a limitation of this study. This may have impacted the ability to detect small but potentially meaningful differences in propulsion or steering performance between interlocking ring design and steering conditions. Consequently, non-statistically significant findings should be interpreted with caution.

For the forward self-propelled motion, the mean slip ratio, $\:{s}_{\text{r}}$, measured in 5-wt% tissue-mimicking phantoms was 0.37 (number of repetitions, *n*, of 6, for the 1R5 up condition) for our needle consisting of six 0.25-mm diameter outer needle segments and a 0.35-mm central needle segment. This value is considerably larger than the slip ratio reported by Scali et al.^14^ for a needle consisting of seven 0.25-mm diameter needle segments connected with a flower-shaped ring evaluated in 4-wt% tissue-mimicking phantoms, which has a mean $\:{s}_{\text{r}}$ of 0.21 (*n* = 5). The different slip ratios can be attributed to the use of a softer tissue-mimicking phantom (4 wt% compared with 5 wt%) by Scali et al.^14^, which contributes to reduced cutting forces at the tip of the needle, leading to a smaller $\:{s}_{\text{r}}$. Our slip ratio is also larger than the slip ratio reported by Scali et al.^13^ for a discrete bevel-tip needle consisting of six 0.25-mm segments connected with a heat shrink tube evaluated in 5-wt% tissue-mimicking phantoms, which has a mean $\:{s}_{\text{r}}$ of 0.28 (*n* = 10). This difference may be explained by the fact that Scali et al.^13^ used fewer needle segments (six compared with seven) than we did, resulting in a smaller overall needle diameter than our needle (0.8 mm compared with 0.89 mm). In our needle, the central needle segment does not contribute to the friction forces that help the self-propelled motion but does increase the cutting forces at the tip of the needle. Previous work confirmed that the peak axial needle insertion force increases with increasing needle size^35^.

For the steering motion, the mean deflection-to-insertion ratio, $\:{d}_{\text{r}}$, for our 1R5 up configuration in 5-wt% tissue-mimicking phantoms was 0.40 (*n* = 6). This value is considerably larger than the deflection-to-insertion ratios reported by Scali et al.^12,14^ for discrete bevel-tip needles with mean $\:{d}_{\text{r}}$ values of 0.0686 (*n* = 5) and 0.097 (*n* = 8). The different deflection-to-insertion ratios indicate more effective steering with a prebent bevel-shaped needle tip than with a discrete bevel tip.

Returning to the source of inspiration for our needle, the parasitic wasp, Cerkvenik et al.^5^ reported a median insertion speed, $\:v$, of 0.73 mm/s for the fruit-fly parasitic wasp (*Diachasmimorpha longicaudata* Ashmead (Hymenoptera: Braconidae)) probing in a gel with an elastic modulus of 36 kPa. This reported speed is comparable to the mean $\:v$ of the Prebent Ovipositor Needle, which is 0.75 mm/s (*n* = 24). Additionally, the wasp demonstrated complex insertion trajectories characterized by multiple bends in different directions, similar to the capabilities of our needle (Fig. 5). However, differences arise when comparing the curvature, $\:\kappa\:$, of the wasp ovipositor and the Prebent Ovipositor Needle. Cerkvenik et al.^5^ reported a median $\:\kappa\:$ of 0.2 mm^−1^ for the parasitic wasp, whereas the Prebent Ovipositor Needle in the 1R5 configuration demonstrated absolute mean $\:\kappa\:$ of 0.023 mm^−1^ (*n* = 6) and 0.026 mm^−1^ (*n* = 6), for steering upward and downward, respectively, indicating the wasp ovipositor achieved much sharper curves than our needle. Nevertheless, when we correct for the diameter of the ovipositor and needle by calculating the dimensionless curvature (i.e., $\:\left|\kappa\:\right|$ multiplied by the ovipositor width or needle diameter), our Prebent Ovipositor Needle in the 1R5 configuration showed larger mean dimensionless curvatures, ranging from 0.010 (*n* = 6) to 0.012 (*n* = 6), than the reported median dimensionless curvature of the parasitic wasp, which is only 0.0060^5^. This indicates that, after correcting for the needle diameter, our needle achieved curvatures that go beyond those attained by the parasitic wasp.

In the current prototype, we developed and evaluated a steerable needle with a prebent tip of a single length. Adebar et al.^36^ demonstrated that an increase in the length of the prebent tip in an articulated-tip needle results in a larger curvature, $\:\kappa\:$. In future work, the achievable $\:\kappa\:$ of the Prebent Ovipositor Needle can be enhanced by increasing the length of the prebent tip. We hypothesize that an increase in the prebent angle and length of the prebent tip results in more pronounced needle bending, leading to a sharper curve, which can be used to control needle steering.

During the experiments, we observed that the needle segments occasionally diverged at the tip. This divergence may be attributed to our interlocking mechanism, which interlocks the needle segments at the indentation rather than at the tip of the needle. The absence of interlocking support at the needle tip can lead to the diverging of the needle segments because of the needle-tissue interaction forces encountered during insertion, compromising the propulsion efficiency, $\:\eta\:$, and the effectiveness of the steering mechanism. Moreover, the divergence may cause potential tissue damage, making it an important research topic for future wasp-inspired needles. This divergence of the needle segments was not observed during the evaluations of the Prebent Ovipositor Needle with a single 5-mm interlocking ring and with two 3-mm interlocking ring. However, needle segment divergence was observed in the preliminary evaluation of the Prebent Ovipositor Needle with a single 3-mm interlocking ring. To address this limitation in future designs, we recommend repositioning the indentations and, thereby, the interlocking ring closer to the needle tip or exploring alternative interlocking designs that provide interlocking support along the entire needle length, from the base to the tip, inspired by the olistheter mechanism of the wasp’s ovipositor.

One of the consequences of needle insertion into soft tissue is tissue displacement^37^. Leibinger et al.^11^ demonstrated that wasp-inspired self-propelled motion can reduce tissue motion and deformation. The outer needle segments continuously push and pull the tissue around them, applying a constant strain to the tissue. As a result, viscoelastic materials such as tissue show stress relaxation, meaning that the internal stress within the material does not remain constant but rather decreases^38^. This stress relaxation may help mitigate the extent of tissue trauma during needle insertion. In future *ex vivo* experiments with our Prebent Ovipositor Needle, tissue damage can be assessed through histological evaluation^39^, allowing for detailed analysis of cellular integrity, tissue morphology, and any potential structural changes induced by the needle insertion.

Our experiments demonstrated the ability of the Prebent Ovipositor Needle to self-propel and steer in tissue-mimicking phantoms. We assessed the steering performance based on the deflection-to-insertion ratio, $\:{d}_{r}$, and the curvature, $\:\kappa\:$. However, for a critical evaluation of the needle’s steering performance in a clinical setting, future studies should assess the endpoint error^40^. Furthermore, image guidance techniques can be used to visualize the trajectory of the needle inside the tissue, and implementing a feedback control mechanism within the actuation system can enable real-time correction of this trajectory. Moreover, we evaluated our needle in a controlled environment using tissue-mimicking phantoms. To develop a complete picture of the propulsion and steering performance of the needle in a clinical setting, *ex vivo* and *in vivo* tissue experiments are required. Previous studies have already successfully demonstrated the ability of similar wasp-inspired needles to self-propel through *ex vivo* tissue^15,16^. These previous studies suggest that our needle can perform similarly.

The needle presented in this study represents just one of many potential designs that can be derived from our steerable needle concept. The number, diameter, and length of the needle segments can be changed to accommodate specific medical applications. Additionally, the central needle segment can be replaced with a hollow tube, enabling tissue sample extraction or the insertion of functional elements, such as an optical fiber for optical biopsy or for focal laser ablation to treat prostate cancer^41^.

## Materials and methods

### Needle fabrication

Our needle prototype comprises seven parallel-positioned nitinol segments, which are superelastic and straight-annealed, and an interlocking ring that constrains needle segment rotation. The interlocking ring was produced out of a stainless-steel capillary tube (outer diameter of 0.89 mm). To create the hexagonal-shaped hole (inradius of 0.32 mm), we used wire Electrical Discharge Machining (EDM). The hexagon has rounded corners with a radius of 0.075 mm, corresponding to the wire radius used in the wire EDM process. The needle segments consist of a single central needle segment with a diameter of 0.35 mm and six outer needle segments with a diameter of 0.25 mm, each with a length of 200 mm.

The needle segments were sharpened by wire EDM to increase the tip’s cutting efficiency and minimize the required insertion force. The straight central needle segment was sharpened to a triangular angle of 20°, whereas the prebent central needle segment was sharpened to an asymmetric bevel angle of 20°. The outer needle segments were sharpened to form lancet-shaped tips with an angle of 20°, similar to previously described wasp-inspired needles^20,29^. In the assembled needle, the sharpened tips of the outer needle segments point toward the center, with their bevels facing outward. Additionally, to accommodate the interlocking ring, 12-mm indentations were created in the outer needle segments by wire EDM, starting 2 mm from the tip of the needle segment. At the location of the indentation, the cross-section of the outer needle segment resembles half a cylinder, with the flat surface facing outward. This results in a radial clearance between the outer needle segments and the inner surface of the hexagonal-shaped hole of the interlocking ring of 20 μm.

In order to create a prebent shape at the tip of the central needle segment, we used a heat treatment method that defines the shape of the superelastic nitinol needle segment while retaining its superelastic properties^42^. The nitinol needle segment was constrained in an aluminum fixture, secured with bolts to achieve a shape featuring a 30° bending angle and an 8-mm curvature length at the needle tip. The thermal treatment involved placing the mold with the nitinol needle segment in a small chamber furnace, where it was heated to a temperature of 550 °C for 20 min. During this process, the needle segment was kept in its deformed shape. Following the heating period, the mold with the needle segment, was rapidly cooled by quenching it in room-temperature water. As a result, the nitinol acquired a prebent austenitic shape at room temperature while retaining its superelastic characteristics.

### Needle control

In order to achieve the required self-propelled motion sequence, the needle was connected to a control and actuation system (Fig. 3A,B). The control system comprises an Arduino board (MEGA 2560) in conjunction with six stepper drivers (Moons’ SR3-MINI). The control system controls the actuation system, which actuates the six outer needle segments to achieve the required self-propelled motion sequence. This actuation system contains six linear actuators (Thomson MLAX8A05-0157S0039-E4-S01, displacement resolution 1.57 μm) that enable the back-and-forth movement of the six outer needle segments. The linear actuators were arranged in a circular configuration to correspond with the positions of the six outer needle segments.

As the linear actuators run at a larger diameter than the six needle segments do, sliders and capillary tubes are used to guide the needle segments toward the central needle segment and ensure that they reach their final insertion diameter. Each linear actuator is connected to a slider that points toward the center (Fig. 3C). The outer portion of the slider is securely clamped to an actuator rod via a set screw. Each outer needle segment is fastened to the central portion of a slider with another set screw, ensuring parallel alignment of the needle segments during actuation. Finally, the capillary tubes further guide the outer needle segments toward the central needle segment and their final insertion diameter. In order to prevent buckling of the needle between the actuation system and the tissue-mimicking phantom, the needle is fed through four capillary tubes (T_1_, T_2_, T_3_, and T_4_) arranged in a telescopic manner. The tube diameters are T_1_ = 2.5 mm, T_2_ = 2.0 mm, T_3_ = 1.6 mm, and T_4_ = 1.2 mm. The lengths of T_1_-T_3_ are 30 mm and that of T_4_ is 40 mm. Within the substrate, the substrate exerts an inward force toward the needle’s centerline on the needle segments, thereby preventing the buckling of the individual needle segments.

### Experimental design

The needle’s self-propelled motion relies on the control and actuation system that sequentially translates the six outer needle segments in seven steps per *actuation cycle*. During *Steps 1–6* (Fig. 3D), each outer needle segment is advanced once by the stroke length in a single step and slowly retracted with respect to the actuation system during five steps, with each retraction step covering one-fifth of the stroke length. When there is no slip, the retracting needle segments remain stationary with respect to the substrate and during each actuation cycle, the outer needle segments are advanced by $\:\frac{6}{5}$ stroke length with respect to the substrate. This continuous motion of the outer needle segments applies a constant strain to the surrounding substrate. The Supplementary Materials show more information on the actuation sequence for wasp-inspired self-propelled needles.

For the tissue-mimicking phantoms, gelatin powder of type Dr. Oetker 1-50-230004 (Dr. Oetker Professional, Amersfoort, The Netherlands) was mixed with water with a gelatin weight ratio of 5 wt%. The gelatin/water mixture was poured into molds and stored overnight at 5 °C for solidification. Afterward, the solidified gelatin/water mixture was cut to its final dimensions with a stamping die (width 40 mm, length 110 mm, height 20 mm) and placed on the cart. Millimeter graph paper attached at the bottom of the cart and the ruler of the air track were used as a reference to measure variables $\:{d}_{\text{m}}$, $\:{d}_{\text{s}}$, and $\:{d}_{\text{d}}$ during needle propulsion with an approximative accuracy of 0.5 mm. To ensure the repeatability of the measurement method, the experimental setup was not moved in between the measurements.

For each measurement, a new gelatin phantom was placed on the cart. Before each measurement, the Prebent Ovipositor Needle was inserted over an initial distance of 30 mm inside the tissue-mimicking phantom to ensure initial contact between the needle segments and the phantom. During every measurement, a camera, vertically positioned on a tripod, captured the position of the needle inside the tissue-mimicking phantom to measure variables $\:{d}_{\text{m}}$, $\:{d}_{\text{s}}$, and $\:{d}_{\text{d}}$. Every measurement was performed with an actuated speed of 10 mm/s for the advancing needle segments, the stroke length, $\:S$, set to 2 mm, and the number of actuation cycles, $\:C$, set to 25. Each condition was repeated six times.

### Data analysis

To statistically compare our evaluated conditions (i.e., 1R5 forward versus 1R5 down, 1R5 down versus 1R5 up, and 1R5 up versus 2R3 up) for $\:\eta\:$ and $\:{d}_{\text{r}}$, Mann-Whitney U tests with exact two-tailed p-values were performed. Due to the small sample sizes (*n* = 6 per condition) and the presence of non-normal distributions in one of the dependent variables for one condition (i.e., $\:\eta\:$ for 1R5 up), Mann–Whitney U tests were chosen for all pairwise condition comparisons. This type of nonparametric test does not assume normality and is more appropriate and robust for small samples with potential parametric assumption violations. This led to a total of six statistical comparisons. To control the risk of Type I error from multiple comparisons, a Bonferroni correction was applied, resulting in an adjusted significance threshold of α = 0.0083 (0.05 divided by six tests). Only p-values below this threshold were considered statistically significant. The analysis was done in IBM SPSS Statistics 29.0.0.

## Supplementary Information

Below is the link to the electronic supplementary material.

Supplementary Material 1

Supplementary Material 2

Supplementary Material 3

Supplementary Material 4

## Data Availability

All data needed to support the conclusions of this manuscript are included in the main text, the Supplementary Materials, or at the following DOI: 10.4121/da8b9a7b-c274-4c85-b527-00794811bad1.
