# Supplementary material for: Steering strategies for wasp inspired self propelled needles
Source: Sci Rep. 2025 Aug 23;15:31018. doi: 10.1038/s41598-025-15031-7 (PMC12374996; doi:10.1038/s41598-025-15031-7)
Supplement: Supplementary file 1 — Supplementary Material 1 [file 41598_2025_15031_MOESM1_ESM.docx]

Supplementary Materials:

Efficiency computation

# 1. Wasp-inspired self-propelled needles

In the scientific literature, wasp-inspired needle designs that can be advanced through the tissue without applying an external push force have been proposed. The needles consist of multiple parallel segments that can slide with respect to each other. A bundling mechanism holds the needle segments together at the tip to limit the diverging of the needle segments. The self-propelled motion is achieved by counterbalancing the cutting and friction force of the advancing segments with the friction force generated by the stationary or retracting segments [1]. To achieve the self-propelled motion of the needle, Eq. S1 must hold.

| $\sum_{i=1}^{a} \left( \mathbf{F}_{\mathrm{fric},i}+\mathbf{F}_{\mathrm{cut}, i} \right)\leq\sum_{j=1}^{r} \left( \mathbf{F}_{fric,j} \right)$ | (S1) |
| --- | --- |

Where $a$ is the number of advancing needle segments, $r$ is the number of stationary or retracting needle segments, and $\mathbf{F}_{\mathrm{fric}}$ and $\mathbf{F}_{\mathrm{cut}}$ are the friction and cutting force, respectively. For the self-propelled motion to occur, the friction force of the stationary or retracting needle segments with the surrounding tissue should be equal to the sum of the friction and cutting forces of the advancing needle segments with the surrounding tissue, which can be achieved by keeping the number of advancing needle segments smaller than the number of stationary or retracting needle segments ($a<r$) amongst others. This way, the stationary or retracting needle segments remain stationary with respect to the tissue, whilst the advancing segment moves forward into the tissue. Assuming equal friction between all needle segments and the surrounding tissue, the wasp-inspired self-propelled needle requires at least three parallel needle segments, of which two segments either remain stationary or retract, while one segment advances to achieve self-propulsion.

# 2. Actuation mode

The needle segments of wasp-inspired needles in the scientific literature are actuated using one of the following two actuation modes: 1) step-by-step motion and 2) continuous motion. In the first actuation mode, called the *step-by-step motion* (Fig. S1), the needle segments advance one by one over a pre-defined distance, called the *stroke*. Once all needle segments have reached that distance, they simultaneously retract over the same stroke distance. The two-phase motion sequence, which involves advancing the needle segments one by one followed by retracting all needle segments simultaneously, is referred to as a *cycle*. In the scientific literature, several needle designs were actuated following the step-by-step motion sequence [2-4].

In the second actuation mode, called the *continuous motion* (Fig. S2), the needle segments move simultaneously and are always in motion. In this motion sequence, one needle segment advances over the stroke distance, while the other needle segments retract. For a needle consisting of three needle segments, this means that during every stage of the motion, one needle segment advances over the stroke distance while the remaining two segments retract over half the stroke distance. During one cycle, all needle segments have advanced over the stroke distance in one stage and retracted over the stroke distance in multiple stages. In the scientific literature, several needle and tissue transport designs were actuated following the continuous motion [5-8] (and the first additional experiment in [2]).

**
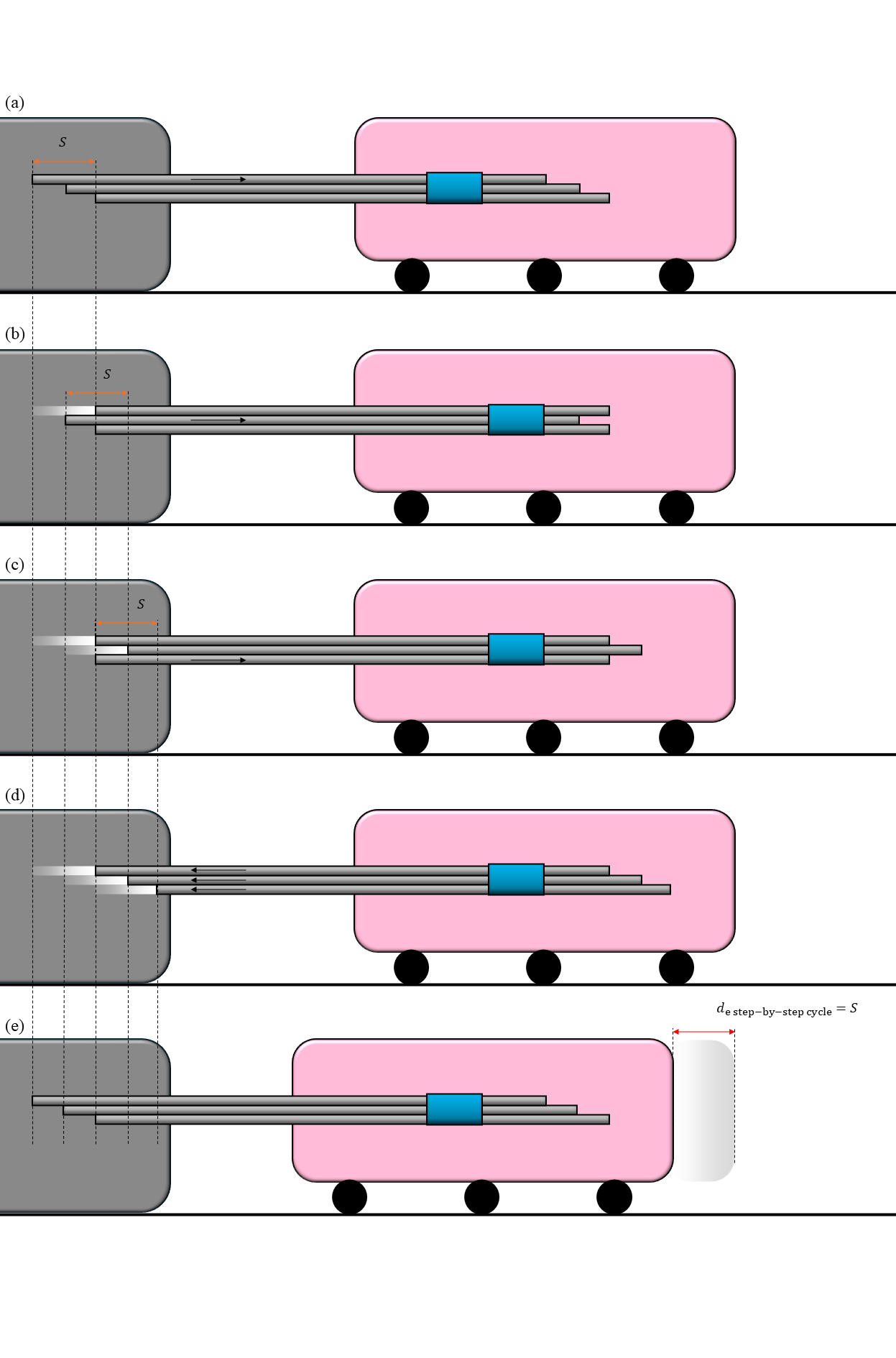
**

**Figure S1. Visualization of the step-by-step motion sequence for a needle consisting of three parallel needle segments.** The visualization shows the needle segments (in light gray), bundled by a bundling mechanism (in blue), the actuation system (in dark gray), and the tissue on a low-friction cart (in pink). For the self-propelled motion, we assume that the retracting and stationary needle segments remain stationary with respect to the tissue. $S$ is the stroke distance, over which the needle segments are advanced and $d_{e step-by-step cycle}$ is the expected propulsion distance of the needle after one cycle by following the step-by-step motion sequence, which is equal to the stroke distance. The rows show the subsequent steps in the step-by-step motion cycle. (a) Segment 1 moves forward over the stroke distance. (b) Segment 2 moves forward over the stroke distance. (c) Segment 3 moves forward over the stroke distance. (d) All segments are retracted over the stroke distance. (e) Final position after one cycle.


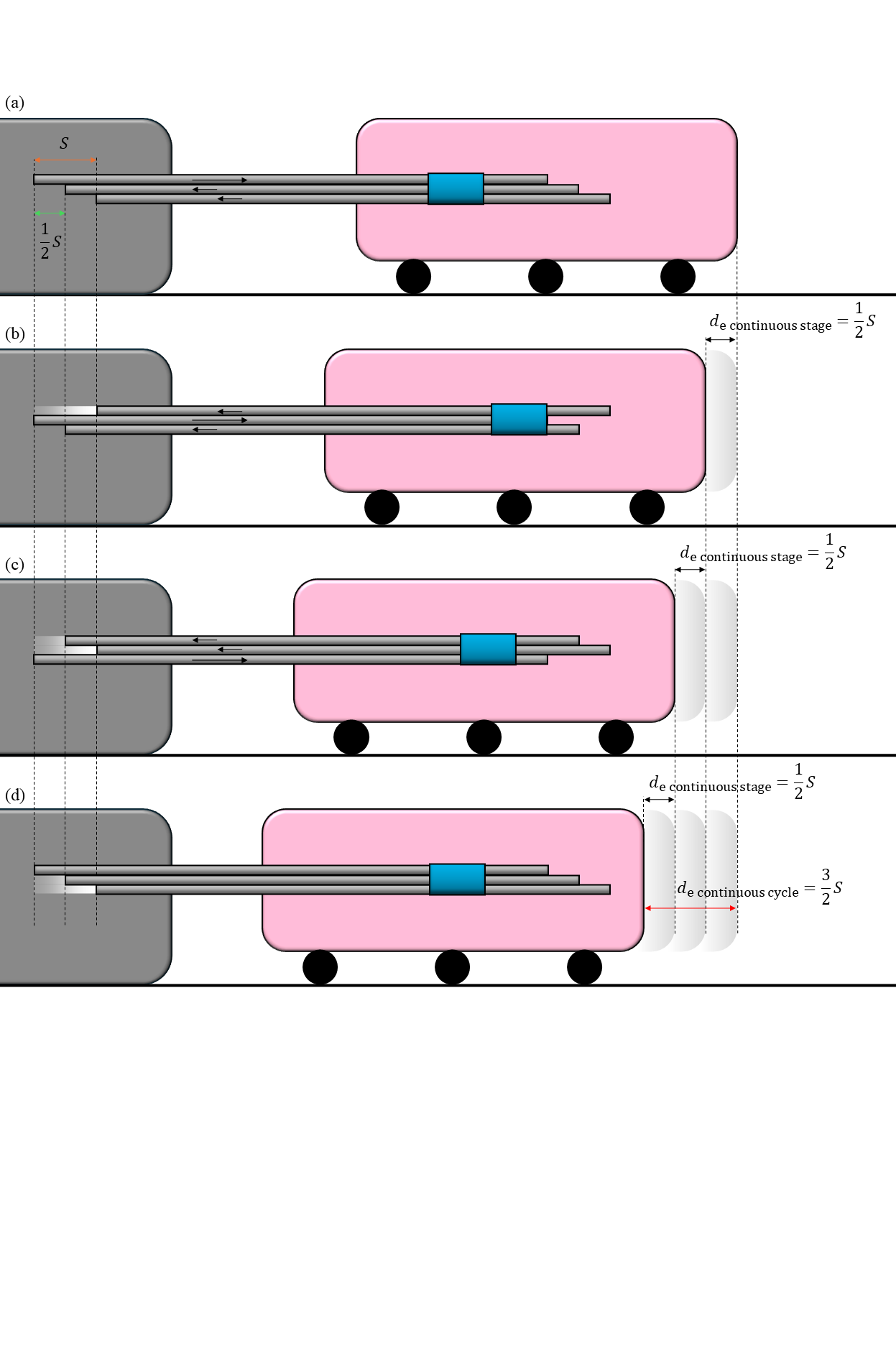


**Figure S2. Visualization of the continuous motion sequence for a needle consisting of three parallel needle segments.** The visualization shows the needle segments (in light gray), bundled by a bundling mechanism (in blue), the actuation system (in dark gray), and the tissue on a low-friction cart (in pink). For the self-propelled motion, we assume that the retracting needle segments remain stationary with respect to the tissue. $S$ is the stroke distance, over which the needle segments are advanced per cycle, $d_{e continuous stage}$ is the expected propulsion distance of the needle after one stage of the cycle by following the continuous motion sequence, and $d_{e continuous cycle}$ is the expected propulsion distance of the needle after one cycle by following the continuous motion sequence, which is equal to 3/2 times the stroke distance. The rows show the subsequent stages in the continuous motion cycle. (a) Segment 1 moves forward over the stroke distance, whilst Segments 2 and 3 move backward over half of the stroke distance. (b) Segment 2 moves forward over the stroke distance, whilst Segments 1 and 3 move backward over half of the stroke distance. (c) Segment 3 moves forward over the stroke distance, whilst Segments 1 and 2 move backward over half of the stroke distance. (d) Final position after one cycle.

# 3. Propulsion efficiency

In order to determine the slip ratio or the efficiency of the needle propulsion, the amount of slip between the needle segments and the tissue (phantom) can be measured. The propulsion efficiency can be computed by dividing the measured propulsion distance of the needle, $d_{m}$, by the expected propulsion distance of the needle, $d_{e}$, as in Eq. S2.

| $\eta=\frac{d_{m}}{d_{e}} \cdot100\%$ | (S2) |
| --- | --- |

The slip ratio of the needle can be calculated as in Eq. S3.

| $s_{\mathrm{ratio}}=1-\left( \frac{d_{m}}{d_{e}} \right)$ | (S3) |
| --- | --- |

The expected propulsion distance of the needle per cycle depends on the actuation mode and the stroke length. For the step-by-step motion, the expected propulsion distance of the needle per cycle is equal to the stroke length ($d_{e step-by-step cycle}=S$). However, for the continuous motion, the expected propulsion distance of the needle per cycle depends on the stroke length and on the number of simultaneously advancing and retracting needle segments during one stage of the cycle, as in Eq. S4. Here, we assume that the retracting needle segments remain stationary with respect to the tissue and the advanced segment moves forward. Therefore, for continuous motion, the expected propulsion distance of the needle per cycle exceeds the stroke length, as in Eq. S5.

| $d_{e continuous stage}=\left( \frac{a}{r} \right) \cdot S$ | (S4) |
| --- | --- |
| $d_{e continuous cycle}=\left( \frac{n}{r} \right) \cdot S$ | (S5) |

Consequently, for continuous motion, increasing the number of needle segments decreases the ratio of the total number of needle segments (i.e., $a+r=n$) to the number of retracting needle segments (i.e., $r$) and, therefore, decreases the expected propulsion distance. More specifically, the expected propulsion distance divided by the stroke over the total number of needle segments will approach a horizontal asymptote at $\frac{d_{e continuous cycle}}{S}=1$ (Fig. S3). The expected propulsion distance per cycle for continuous motion approaches the expected propulsion distance per cycle for step-by-step motion, which is equal to the stroke distance if the number of needle segments increases whilst keeping the advancing needle segments at one.

# 4. Expected velocity

During the insertion of a needle in tissue, the velocity of the needle impacts tissue deformation and damage. Mahvash *et al.* [9] showed that maximizing the needle velocity minimizes tissue deformation and damage and, consequently, results in less needle insertion position error. For wasp-inspired self-propelled needles, the expected needle velocity ($v_{N e}$) and therefore also the actual insertion velocity of the needle depends on the actuation mode, the number of parallel needle segments, and the actuation velocity of the individual needle segments ($v_{S}$). Given the same actuation velocity of the individual needle segments and the same number of needle segments, the expected needle velocity for the step-by-step motion (Eq. S6) is lower than that for the continuous motion (Eq. S7), which is caused by the lower expected actuation efficiency for the step-by-step motion (Eq. S8) compared to that of the continuous motion (Eq. S9).


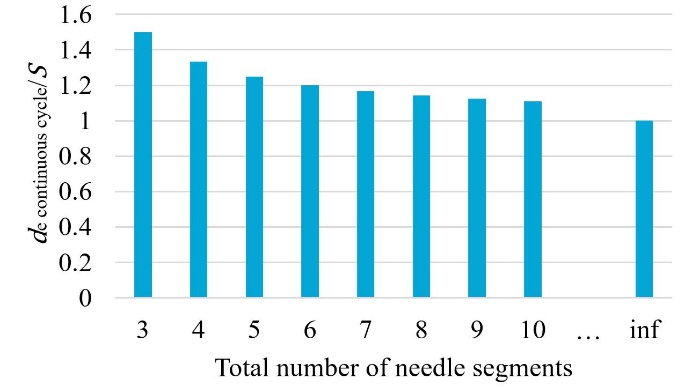


**Figure S3. The expected propulsion distance divided by the stroke distance for the continuous motion.** $d_{e continous cycle}$ is the expected propulsion distance per cycle for continuous motion, $S$ is the stroke distance over which the needle segments are advanced per cycle, and inf means an infinite number of needle segments. When the total number of needle segments increases while keeping the number of advancing needle segments at one, the expected propulsion distance per cycle over the stroke approaches a horizontal asymptote at $\frac{d_{e continuous cycle}}{S}=1$.

| $v_{N e step-by-step}=\frac{\Delta x}{\Delta t}=\frac{d_{e step-by-step cycle}}{t_{n+1}-t_{0}}=\frac{S}{\left( n+1 \right)\cdot\frac{S}{v_{S}}}=\frac{v_{S}}{n+1}$ | (S6) |
| --- | --- |
| $v_{N e \mathrm{continuous}}=\frac{\Delta x}{\Delta t}=\frac{d_{e continuous cycle}}{t_{n}-t_{0}}=\frac{\frac{n}{r}S}{n\cdot\frac{S}{v_{S}}}=\frac{v_{S}}{r}$ | (S7) |
| $\eta_{A e step-by-step}=\frac{1}{n+1}\cdot100\%$ | (S8) |
| $\eta_{A e continuous}=\frac{1}{r}\cdot100\%$ | (S9) |

Where $d_{e step-by-step cycle}$ is the expected propulsion distance of the needle per cycle for the step-by-step motion, $d_{e continuous cycle}$ is the expected propulsion distance of the needle per cycle for the continuous motion, $t$ is the time at a certain step or stage in the motion cycle, $S$ is the stroke distance, $n$ is the total number of needle segments, and $r$ is the number of retracting needle segments.

# References

1 Scali, M. *Self-propelling needles: From biological inspiration to percutaneous interventions*, Delft University of Technology, (2020).

2 Scali, M., Breedveld, P. & Dodou, D. Experimental evaluation of a self-propelling bio-inspired needle in single-and multi-layered phantoms. *Scientific reports* **9**, 1-13 (2019). <https://doi.org:10.1038/s41598-019-56403-0>

3 Scali, M., Kreeft, D., Breedveld, P. & Dodou, D. in *Proceedings of SPIE* Vol. 10162 *Bioinspiration, Biomimetics, and Bioreplication 2017* 1016207 (International Society for Optics and Photonics, Portland, Oregon, United States, 2017).

4 Bloemberg, J., Hoppener, B., Coolen, B., Sakes, A. & Breedveld, P. Design and evaluation of a pneumatic actuation unit for a wasp-inspired self-propelled needle. *PLoS ONE* **19**, e0306411 (2024). <https://doi.org:10.1371/journal.pone.0306411>

5 Bloemberg, J., Trauzettel, F., Coolen, B., Dodou, D. & Breedveld, P. Design and evaluation of an MRI-ready, self-propelled needle for prostate interventions. *PLoS ONE* **17**, e0274063 (2022). <https://doi.org:10.1371/journal.pone.0274063>

6 Sakes, A. *et al.* Development of a novel wasp-inspired friction-based tissue transportation device. *Frontiers in Bioengineering and Biotechnology* **8**, 575007 (2020). <https://doi.org:10.3389/fbioe.2020.575007>

7 de Kater, E. P., Sakes, A., Bloemberg, J., Jager, D. J. & Breedveld, P. Design of a flexible wasp-inspired tissue transport mechanism. *Frontiers in Bioengineering and Biotechnology* **9**, 782037 (2021). <https://doi.org:10.3389/fbioe.2021.782037/full>

8 Bloemberg, J., van Wees, S., Kortman, V. G. & Sakes, A. Design of a wasp-inspired biopsy needle capable of self-propulsion and friction-based tissue transport. *Frontiers in Bioengineering and Biotechnology* **12**, 1497221 (2025). <https://doi.org:10.3389/fbioe.2024.1497221>

9 Mahvash, M. & Dupont, P. E. in *2009 IEEE International Conference on Robotics and Automation.* 3097-3102 (IEEE).
